# A comparative study of various statistical and machine learning models for predicting restaurant demand in Bangladesh

**DOI:** 10.1371/journal.pone.0325449

**Published:** 2025-06-04

**Authors:** Md Shakhawath Hossain, Farjana Parvin

**Affiliations:** 1 Department of Industrial Engineering and Management, Khulna University of Engineering & Technology, Khulna, Bangladesh; 2 Department of Industrial Engineering and Management, Khulna University of Engineering & Technology, Khulna, Bangladesh; Ataturk University, TÜRKIYE

## Abstract

Precise demand forecasting has become crucial for merchants due to the growing complexity of client behavior and market dynamics. This allows them to enhance inventory management, minimize instances of stock outs, and enhance overall operational efficiency. In Bangladesh, there is a significant lack of emphasis on demand forecasting to enhance corporate performance. In recognition of these difficulties, the study seeks to produce predictions by employing two statistical models and three machine learning models. The historical sales data was obtained from a restaurant in Bangladesh, and five specific products were chosen for the purpose of predicting sales. The models have been rated according to their average score of deviation from the optimal root mean squared error. The Multilayer Perceptron and Random Forest algorithms have attained the top two positions. Statistical models such as simple exponential smoothing and Croston’s method have exhibited superior performance compared to XGBOOST model. This study advances demand forecasting techniques in Bangladesh’s restaurant industry by providing valuable insights, comparing different approaches, and suggesting ways to improve forecast accuracy and operational efficiency, thereby demonstrating the practical relevance and applicability of the research to the reader.

## Introduction

Various important aspects impact future sales, which can be identified by analyzing the sales patterns of total sales in a retail store or for a specific product [1]. Businesses that possess a greater understanding of demand or sales are more likely to achieve success. The primary objective of demand forecasting is to assure the acquisition of the correct quantity of commodities at the exact time and place, while devising the most advantageous production volume and inventory, and improving the supply network for efficient distribution [2].

The prediction of product demand typically relies on the specific attributes of the product and the characteristics of the industry [3]. The connection between the features of a product and its industry influences the nuances of a time series analysis used for forecasting demand [4]. The demand in the retail sector is very variable and subject to frequent fluctuations, posing significant challenges for forecasting. Hence, it is crucial for the retail industry to accurately predict the demand in order to differentiate themselves from rivals, prioritize adaptable customer service, promptness, and strict compliance with delivery deadlines, all while maintaining competitive pricing [5].

Due to all of this reasons, researchers and organizations have been using statistical tools to predict outcomes, aiming to improve current methods or discover new methodologies for forecasting. The moving average is a conventional forecasting approach, although a more advanced version called Autoregressive Integrated Moving Average (ARIMA) has been created in recent years. Ramos et al. [6] used ARIMA and exponential smoothing approaches to evaluate their efficacy in forecasting the retail sales of women’s footwear, characterized by products with repetitive fluctuations in their trends. Exponential Smoothing is one of the powerful traditional or statistical method for forecasting. There exist various forms of exponential smoothing techniques. Kalekar elucidated the distinction between various exponential smoothing method in his study [7]. Taylor [8] employed a seasonal exponential smoothing method to forecast the daily sales of a retailer with notable fluctuations. Croston [9] studied forecasting algorithms for intermittent time series and found that current exponential smoothing methods are not particularly appropriate. Using this discovery as a basis, he created his own technique, which has now become a standard reference point in many analyses. These methods are relatively simple and need minimal ability to compute. However, these conventional or statistical approaches may not consistently yield precise outcomes and necessitate additional computational time. Hence, the quest for alternate approaches remained consistently prevalent.

In the last few years, artificial intelligence has sparked a revolution in various corporate sectors. Machine learning and deep learning models are frequently used for demand prediction. An advantage of machine learning methods is their ability to capture the underlying mechanisms, even when specific parameters lack sufficient information. This functionality allows machine learning algorithms to effectively extract meaningful information and produce accurate forecasts, even in complex situations [10]. However, machine learning methods require more computational resources than statistical approaches, leading to a greater need for computer science expertise in implementation [11]. Due to this specific reason, small organizations or businesses encountered difficulty in utilizing machine learning or deep learning models for demand forecasting. Though recent improvements have significantly reduced the cost of computing power and storage capacity and thus opening up new opportunities for enterprises in the field of forecasting [12]. For businesses to transition from traditional statistical methods to machine learning for forecasting, the latter must demonstrate superior predictive accuracy. To achieve this goal, comparing statistics and machine learning models is essential.

Several studies have compared different machine learning models for demand forecasting across various industries. In Moroff et al.‘s work [13], various statistical and machine learning models were employed to make forecasts based on a dataset. The study’s findings indicate that the Multilayer Perceptron has surpassed all other methods in performance. However, statistical models such as Triple Exponential Smoothing have yielded better results compared to XGBOOST and random forest. Makridakis et al. [11] have also conducted similar research. The study conducted a comparison of various statistical methods, such as naïve, moving average, simple exponential smoothing, as well as other machine learning methods including random forest and multilayer perceptron. According to the study, the multilayer perceptron and random forest algorithms outperformed the naïve, moving average, and basic exponential smoothing methods in terms of Root Mean Squared Scaled Error. The research conducted by Deng et al. [14] demonstrates that XGBOOST is the most effective approach for predicting electricity consumption. While Ahmed et al. [15] demonstrated that random forest has given better results than traditional methods. Similar work has also been done by researchers for other areas. Demirsoy et al. [16] have used three different machine learning models to identify drug–drug interactions (DDIs), and according to their study, XGBOOST has performed the best with an accuracy of 78%. XGBOOST has outperformed other models in the study of Asselman et al. [17] as well, where they have used machine learning approaches to predict student performances. Guo et al. [18] also used the XGBOOST-based model for evaluating physical fitness.

In recent years, a number of research studies have been conducted to determine the most practical forecasting method for different sectors in Bangladesh. Arif et al. [19] did a study using various machine learning algorithms to forecast a shop’s demand. The researchers utilized machine learning methods including K Nearest Neighbor, Gaussian Naïve Bayes, and Decision Tree to make predictions. Based on their findings, Gaussian Naïve Bayes achieved the highest accuracy rate of 58.92%. In a study by Suraiya et al. [20], time series forecasting models were used to identify accurate approaches for predicting the demand for printing paper within a specific time frame. Their analysis revealed that using a linear trend equation was the most effective method. Another research by Halder et al. [21] found that the weighted moving average method had the highest accuracy in forecasting the demand for jute products in Bangladesh, with a Mean Absolute Percentage Error of 16.29%. According to Hasan et al. [22], the Holt-Winter Multiplicative Forecasting Method outperforms other techniques when applied to real-world data sets from a clothing manufacturer. Additionally, Hasin et al. [23] studied the demand within the retail trade sector in Bangladesh and found that the Artificial Neural Network outperforms the Holt-Winters technique, with a Mean Absolute Percentage Error of 10.1% compared to the Holt-Winters technique’s 29.1%.

Machine learning methods have the potential to enhance demand forecasting by effectively managing complex relationships among various causal factors, including non-linear patterns that influence demand [24]. The effectiveness of machine learning methods relies heavily on the availability of a sufficient amount of data, as they do not primarily rely on assumptions about the data [25]. Obtaining adequate sales data in a country like Bangladesh is extremely challenging. Many restaurants avoid storing all their data, and those that do are hesitant to provide it for research purposes. These constraints have resulted in a lack of research specifically focused on identifying the best forecasting methods suitable for the restaurant or food industry in Bangladesh.

This study seeks to fill this gap by applying machine learning models to forecast future sales of a Bangladeshi restaurant. The study employs k-fold validation and Monte Carlo simulation to enhance the robustness of the results, which ensures reliable performance evaluations. Additionally, the study provides a thorough analysis and comparison of statistical and machine learning models, which can help restaurant owners to decide whether to invest in computational resources for machine learning or not.

## Forecasting models

### Statistical models

#### Simple exponential smoothing.

Exponential Smoothing is a forecasting technique that accurately predicts discrete time series data quickly and efficiently [26]. It involves creating weighted averages of past data, with the weights decreasing exponentially over time and giving greater importance to the most recent observations [27,28]. Simple Exponential Smoothing is especially valued for its quick calculations and easy usability. It is ideal for predicting future values in real-time or when data is scarce [29]. The equations for simple exponential smoothing are as follows:


$${\hat{y}}_{t}=\;\alpha y_{t-1}+(1-\alpha ){\hat{y}}_{t-1}$$ (1)

In time series analysis, the symbol α represents the smoothing parameter. α is a value between 0 and 1. A smaller α places more weight on past observations, while a larger α emphasizes the importance of the current observation. By adjusting the smoothing parameter, the forecaster can choose the data approximation method and remove noise from the data.

#### Croston’s method.

Traditional forecasting models, like simple exponential smoothing, can occasionally prove ineffective for intermittent demands because intermittent demand patterns correlate demand arrivals with demand size [30]. Croston’s method, a popular technique for forecasting intermittent demand, can address these issues. It separately applies simple exponential smoothing to the intervals between nonzero demands and their sizes [9]. Following each demand event, it updates the smoothed estimates of the average demand size and the average interval between demands. If no demand occurs, the estimates remain unchanged [31].

The algorithm for Croston’s method can be outlined as follows [32]

If Z_t _= 0, then


$${\hat{Z}}_{t}=\;{\hat{Z}}_{t-1}$$ (2)

$$\hat{P_{t}}=\;{\hat{P}}_{t-1}$$ (3)

$$q_{t}=\;q_{t-1}+1$$ (4)

If Z_t _> 0, then


$${\hat{Z}}_{t}=\;{\hat{Z}}_{t-1}+\;\alpha (Z_{t}-\;{\hat{Z}}_{t-1})$$ (5)

$${\hat{P}}_{t}=\;{\hat{P}}_{t-1}+\;\alpha (P_{t}-\;{\hat{P}}_{t-1})$$ (6)

$$q_{t}=1$$ (7)

where,

Z_t_ – (non-zero) demand at time t,

${\hat{Z}}_{t}$ – forecast of the (non-zero) demand in period t,

${\hat{X}}_{t}=\;\frac{{\hat{Z}}_{t}}{{\hat{P}}_{t}}$ – (average) demand per period,

$X_{t}$ – empirical sales in period t (both zero and non-zero demand),

P_t_ – number of time periods between two non-zero sales,

${\hat{P}}_{t}$ – forecast of demand interval in period t,

q – number of periods since the last non-zero sales,

α ∈ (0, 1) – smoothing constant value.

### Machine learning models

#### Extreme Gradient Boosting (XGBOOST).

XGBOOST, short for extreme gradient boosting, combines two weak models to create a stronger one. It efficiently handles large datasets quickly and effectively using machine learning techniques based on the gradient boosting architecture [33]. We can describe XGBOOST as follows: given a dataset D = [x, y] with n observations, where x represents the independent variables and y is the dependent variable, we iterate a specified number of times, denoted by K.

Function B can be used to predict the outcome, with ŷ representing the forecast for the i-th sample at the b-th boost. Moreover, f can signify the construction of a tree-like structure q, where each leaf j is assigned a weight score wj. The final prediction for a specific sample xi is calculated by summing the scores from all branches, as depicted in equation 4 [34].


$$\hat{y}=\;\sum_{b=1}^{B}f_{b}\left(x_{i}\right)$$ (8)

#### Random forest.

The Random Forest algorithm entails constructing a collection of decision trees and then combining the predictions made by each tree [35]. This method involves creating numerous decision trees and summarizing their results based on the training sample set [10]. Each tree in the forest depends on the values of a randomly selected vector that is independent and identically distributed among all trees. To use this approach, it is necessary to define the characteristics of the models, such as the number of trees to be created and the number of variables to be randomly chosen as candidates at each split [36]. The Random Forest algorithm showcases improved resilience to noise and reduced vulnerability to overfitting on the training dataset by combining the predictions of many Decision Trees [37].

#### Multilayer perceptron.

The multilayer perceptron (MLP) is a specific type of neural network. It uses a design called feed-forward. The MLP consists of three layers: the input layer, the hidden layer, and the output layer. The input layer receives the input data, the hidden layer processes the data, and the output layer presents the results. The number of hidden layers determines the network’s depth [38,39]. The hidden layer, located between the input and output layers, is the main computation center of the neural network, while the output layer performs the regression or classification procedure.

## Methods

This study employs and compares statistical and machine learning models to forecast restaurant sales in Dhaka, Bangladesh. Simple Exponential Smoothing and Croston’s Method were used for statistical forecasting, capturing trends and intermittent demand, respectively. Machine learning models—Extreme Gradient Boosting (XGBOOST), Random Forest, and Multilayer Perceptron (MLP)—were implemented to improve predictive accuracy.

### Data collection and processing

Sales data was collected from a restaurant named ‘Sugar N Spice’ located in Dhaka, Bangladesh. The collected data includes sales statistics for over 20 products over 15 months, from June 2022 to August 2023. The raw data also included the discounts offered for each product over time. 5 products have been selected for further investigation: Noodles, Chicken, Hot Coffee, Rice, and Soup. Additional features, including Customer Purchasing Index (CPI), inflation rate, average monthly temperature, average monthly rainfall, national holidays, and festive months, were incorporated for model enhancement. The data of CPI and inflation rate has been imported from publicly available monthly statistics of the ‘Bangladesh Bureau of Statistics’ [40]. The average monthly temperature and rainfall data has been imported from the publicly available data of the ‘Bangladesh Meteorological Department’ [41]. With only 15 months of data and no missing values, no data preprocessing was necessary.

### Implementation

Machine learning models were developed using Python 3.10, leveraging libraries including Scikit-learn (1.2.2), Pandas (1.5.3), NumPy (1.24.3), and Matplotlib (3.7.1). All scripts were executed in the Google Colaboratory environment. Model robustness was ensured through 5-fold cross-validation (k = 5), with four subsets utilized for model training, while the remaining subset was employed for testing in each iteration. Manual hyperparameter tuning was performed to define optimal values for the model and these parameters are displayed in **Table 1**.

**Table 1:** Parameter employed for different models.

Forecasting Method	Parameter Setting
XGBOOST	objective: ‘reg:squarederror’ n_estimators: 100 max_depth: 5 learning_rate: 0.1 random_state: 42
Random Forest	n_estimators: 100 max_depth: 10 random_state: 42
Multilayer Perceptron	hidden_layer_sizes: (64, 32) max_iter: 1000 random_state: 42 activation: ‘relu’ solver: ‘adam’ learning_rate: ‘constant’ alpha: 0.0001

To quantify forecast uncertainty, Monte Carlo Simulation with 1,000 iterations was implemented. Gaussian noise (mean = 0, standard deviation = 0.05) was added to the test inputs using NumPy, generating perturbed datasets for each iteration. These perturbed inputs were passed through a trained model using Scikit-learn, and the resulting prediction distributions were used to compute the mean forecast and standard deviation, representing the predictive uncertainty.

Simple Exponential Smoothing was computed manually utilising Equation 1. The initial prediction was established based on the first actual data point, and the following forecasts were computed iteratively using the formula.

Croston’s method was implemented in Python without relying on external time series forecasting libraries. The implementation involved decomposing the time series into demand sizes and intervals, then applying exponential smoothing separately to both components before reconstructing the forecast.

### Visualisation of data

Data trends and model predictions were visualised using Matplotlib (version 3.7.1). Line plots illustrated sales trends over time, while heatmaps represented correlation matrices to analyse feature relationships. Line plots were also used to illustrate actual versus predicted sales over time for each product, providing a clear visual comparison of the model’s performance. Uncertainty in the predictions of machine learning models was represented using shaded regions around the forecast lines and bar charts compared forecast accuracy across models. These plots enabled an in-depth assessment of the forecast accuracy and variability.

### Evaluation metrics

Model performance was assessed using Root Mean Squared Error (RMSE) and Mean Absolute Percentage Error (MAPE), which were implemented using the sklearn.metrics module. These metrics provide insights into the accuracy and reliability of the forecasts.

RMSE quantifies the standard deviation of prediction errors, ensuring that the error is presented in the same units as the target variable.

MAPE measures the average percentage error, offering a scale-independent evaluation.

A performance score was computed by normalising each model’s RMSE relative to the lowest RMSE observed per product.

## Results & discussions

### Data analysis

**Fig 1:**
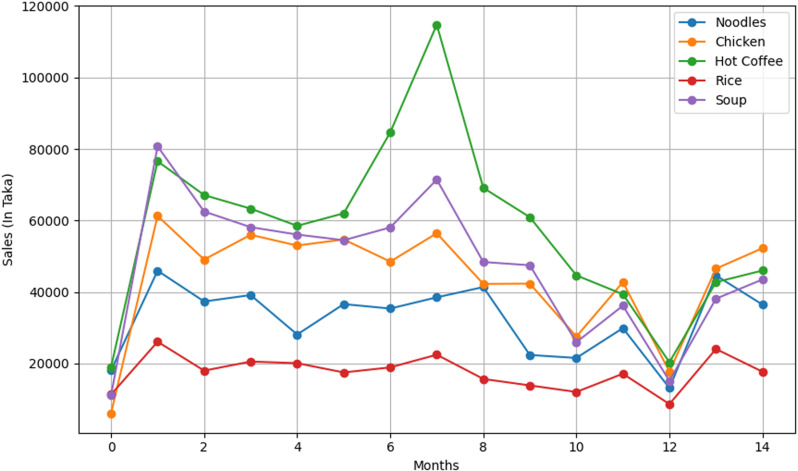
Monthly sales trends for five products over 15 months. The graph displays actual sales amounts (in Bangladeshi Taka) for five products over 15 months, from June 2022 to August 2023.

As shown in **Fig 1**, sales were at their lowest levels in June 2022 within the given period. Sales remained relatively stable throughout the year, with a notable surge in January 2023. This spike, particularly for Hot Coffee, suggests a potential seasonal effect, possibly driven by increased coffee consumption during the winter months. Research indicates that coffee sales fluctuate with seasonal temperature changes [42]. The subsequent decline in sales by June 2023 follows a similar trend to the previous year, likely influenced by Bangladesh’s monsoon climate, which affects consumer behavior and outdoor dining patterns.

**Table 2** presents sales data for five selected products. Hot Coffee exhibits the highest average sales (57,936.08 Taka) and the greatest variability (standard deviation: 24,457.57 Taka), with sales ranging from 19,024.05 to 114,757.15 Taka. The high volatility suggests that demand is significantly influenced by external factors, possibly seasonal variations, weather conditions, and promotional campaigns. This fluctuation has major financial implications for restaurant operations, impacting inventory management, staffing, and pricing strategies. Restaurants must optimize stock levels to prevent wastage during low-demand periods while ensuring adequate supply during peak sales months. A dynamic pricing approach—offering discounts during off-peak months—could help stabilize revenue.

**Table 2:** Statistical Parameters of all products during 15-month period (In Taka).

Statistical Parameters	Noodles	Chicken	Hot Coffee	Rice	Soup
Mean	32566.54	43759.13	57936.08	17599.30	47148.41
Std	9937.26	15397.44	24457.57	4799.87	19489.95
Min	13128.57	6063.09	19024.05	8638.09	11092.86
Max	45947.62	61276.19	114757.15	26085.71	80733.33
25%	25242.86	42300.00	43700.00	14766.67	37142.86
50%	36471.43	48495.24	60919.05	17704.76	48380.95

In contrast, Rice has the lowest average sales (17,599.30 Taka) and a minor variation (standard deviation: 4,799.87 Taka), suggesting consistent and stable demand. Given its predictability, restaurants may not need aggressive marketing efforts for this product, potentially reducing advertising costs. Additionally, stable sales indicate that workforce planning for Rice may require minimal adjustments, unlike volatile products that necessitate hiring extra staff during peak periods and scaling down when demand drops.

Chicken exhibits moderate volatility (standard deviation: 15,397.44 Taka), presenting a unique challenge. Understanding the drivers of this fluctuation could help in devising future strategies for smoother sales forecasting. Further research could explore the interplay between sales trends and customer purchasing behaviors to enhance operational efficiency.

**Fig 2:**
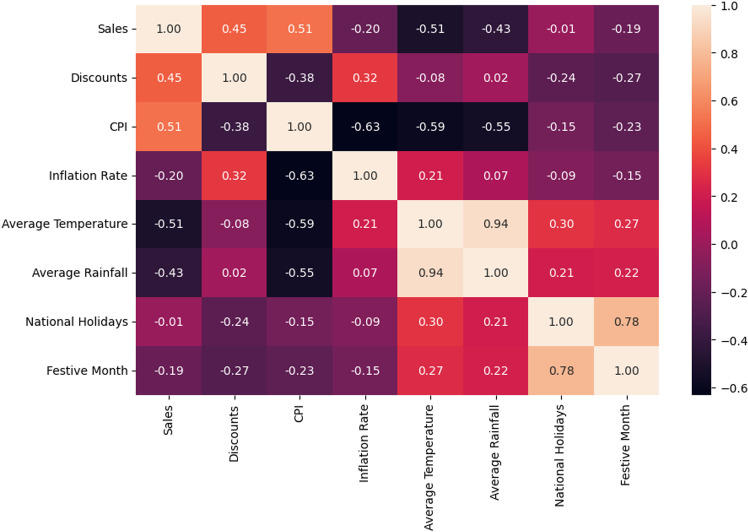
Correlation matrix of total sales and key features. The heatmap shows correlation coefficients between the sum of sales for five products and features like Discounts, CPI, Inflation Rate, Average Temperature, Rainfall, National Holidays, and Festive Months.

**Fig 2** presents the correlation between sales and external factors such as discounts, inflation, and weather conditions. The most significant positive correlations appear between sales and discounts (0.45) and between sales and the Consumer Price Index (CPI) (0.51). While this suggests that price reductions boost sales, it is equally possible that discounts are more likely to be applied during periods of low sales, making it essential to differentiate causation from correlation. On the other hand, sales show a strong negative correlation with both average temperature (−0.51) and average rainfall (−0.43), reinforcing the impact of seasonality on consumer behavior. The decline in sales during the monsoon season could be attributed to lower customer mobility, while the lower demand for hot coffee in warm months aligns with expected seasonal preferences.

### Performance analysis

#### XGBOOST.

**Fig 3:**
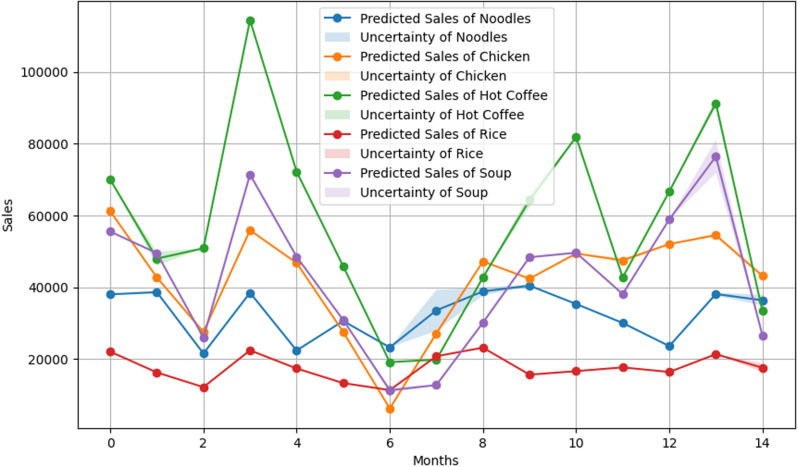
XGBOOST Model Results. Predicted sales and uncertainty bands for the XGBOOST model.

The Mean Absolute Percentage Error for XGBOOST was 56.18%, which is the highest among the machine learning models. XGBOOST successfully captured the sales trend of products that have exhibited lower amounts of sales, like Rice and Noodles. But the model struggled to capture the fluctuations in sales of certain products, such as Chicken, which, on average, increased the MAPE values. Multiple products display the uncertainty bands of this model around the peaks and troughs, as observed in **Fig 3**, indicating variability in the model’s confidence. Although XGBOOST is famous for the robustness of the model, poorer performance in this study suggests that the underlying data require more preprocessing to align the strength of the model.

#### Random forest.

**Fig 4:**
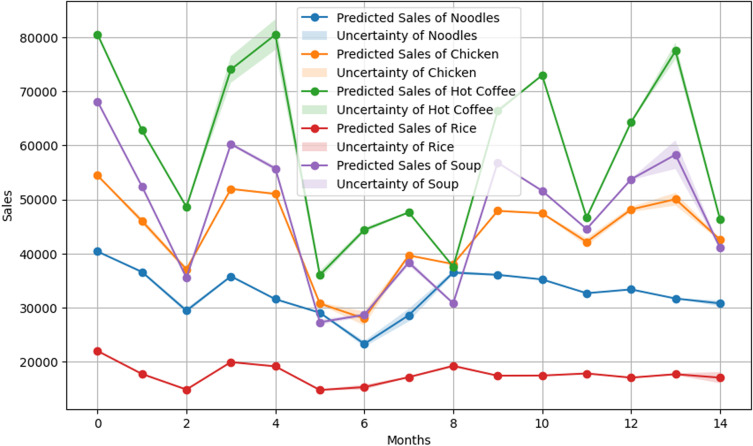
Random Forest Model Results. Predicted sales and uncertainty bands for the random forest model.

The random forest model performed better than the XGBOOST model, with a MAPE of 52.43%. The performance of the model was quite similar to the XGBOOST model, as the random forest also gave the best result for Rice and Noodles but struggled with Chicken. The random forest model demonstrated superior performance in terms of uncertainty. **Fig 4** presents that, the uncertainty bands for the random forest model were narrower than those of the XGBOOST model, which indicates better confidence in prediction. Uncertainty bands in Hot Coffee and Soup identify areas where the model’s performance is less reliable.

#### Multilayer perceptron.

**Fig 5:**
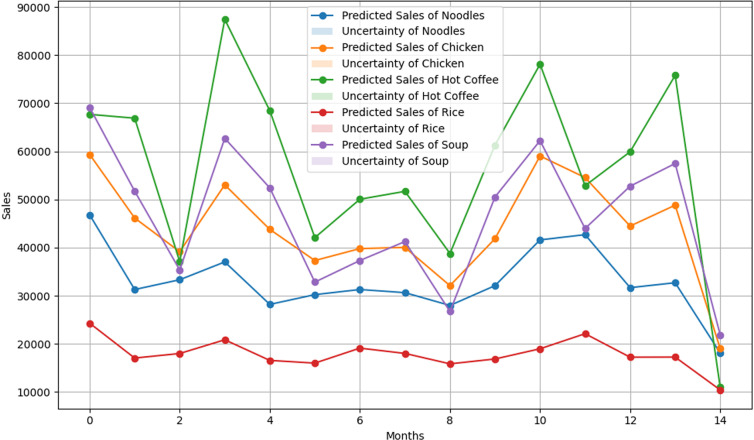
Multilayer Perceptron Model Results. Predicted sales and uncertainty bands for the Multilayer Perceptron (MLP) model.

The multilayer perceptron model has demonstrated the best performance with a MAPE of 46%. Although the multilayer perceptron has outperformed the other two models, it has also followed the same trend, exhibiting the best performance for Rice and the worst performance for Soup. However, the multilayer perceptron demonstrates a higher level of confidence in its predictions compared to the XGBOOST and random forest models. The uncertainty bands for multilayer perceptron were much narrower and almost invisible in **Fig 5**.

**Fig 6:**
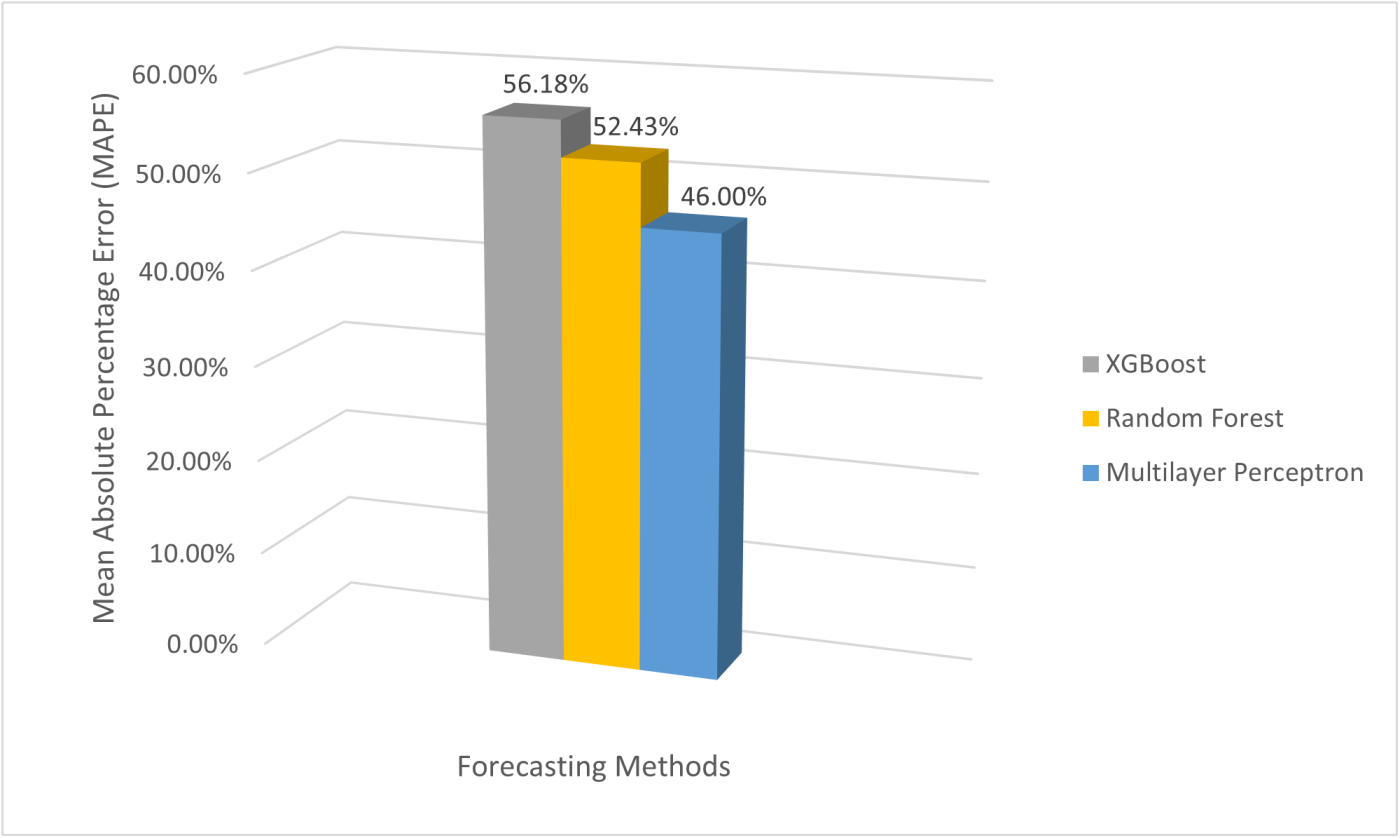
Average Mean Absolute Percentage Error (MAPE) of different forecasting methods. The bar chart compares the MAPE values of forecasting methods. Multilayer Perceptron achieves the lowest MAPE (46.00), followed by Random Forest (52.43), and XGBOOST (56.18).

Overall, multilayer perceptron is the best performer among the three machine learning models, as depicted by MAPE value in **Fig 6**. But all the models have struggled to predict the sales of products with variable patterns, particularly for Chicken. However, all the models performed better for products with less variability and lower sales, suggesting that they need more data to accurately capture the seasonal sales pattern. The uncertainty bands also highlight the challenges posed by the volatility of product sales.

#### Comparative analysis.

The table presents the root mean squared error for all products of all models (**Table 3**). The multi-layer perceptron model performed the best for all of the chosen products.

**Table 3:** Root Mean Squared Error of Forecasting Models by Product.

	Statistical Model		Machine Learning Model		
Product	Simple Exponential Smoothing	Croston Method	XGBOOST	Random Forest	Multilayer Perceptron
Noodles	13050.18	12761.06	13539.67	11794.75	11441.26
Chicken	23704.90	24132.10	21506.02	17140.62	15562.67
Hot Coffee	38946.76	30445.22	26473.61	21734.15	18134.17
Rice	7362.34	6253.81	6857.65	5774.08	5151.99
Soup	31322.03	26338.50	22889.98	21221.87	17271.36

**Fig 7:**
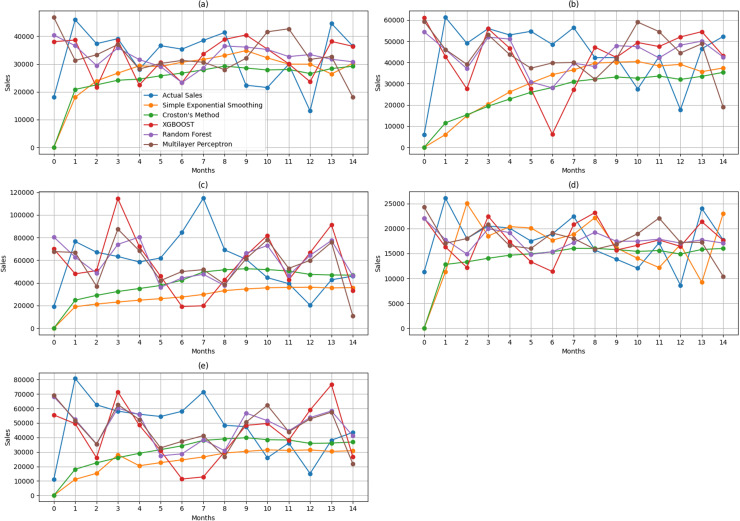
Actual Sales vs. Predicted Sales using five forecasting models. The figure presents a comparison of actual sales and forecasted sales for five products over 15 months using XGBOOST, Random Forest, Multilayer Perceptron, Simple Exponential Smoothing, and Croston Method. This figure is a multi-graph comparison, where (a) represents Noodles, followed by (b) Chicken, (c) Hot Coffee, (d) Rice, and (e) Soup.

Different models have given the best results for different products. From **Fig 7** it can be seen that, the precision of the models varies for each product and depends on the specific attributes of the data. To better understand the performance, it’s essential to determine how close each model is to the optimal performing model. The performance score for this specific objective is displayed in **Table 4**. A higher numerical value indicates that the model is more suitable across a broader range of products. The ranking of the models is determined by calculating the average performance score.

**Table 4:** Performance scores of Forecasting Models.

	Statistical Model		Machine Learning Model		
Product	Simple Exponential Smoothing	Croston Method	XGBOOST	Random Forest	Multilayer Perceptron
Noodles	87.67%	89.66%	84.50%	97%	100%
Chicken	65.65%	64.49%	72.36%	90.79%	100%
Hot Coffee	46.56%	59.56%	68.50%	83.44%	100%
Rice	69.98%	82.38%	75.13%	89.23%	100%
Soup	55.14%	65.57%	75.45%	81.38%	100%
Average	65%	72.33%	75.19%	88.37%	100%

Table 4 shows that the Multilayer Perceptron model consistently outperforms other models across all performance metrics. Multilayer Perceptron is famous for capturing complex data patterns and accurate predictions. The study yielded similar results, with MLP achieving the best results in both RMSE and MAPE values. So MLP can be an excellent choice for forecasting across different sectors and industries.

Although random forest has given less accurate results than multilayer perceptron, it can be a beneficial choice as well, as this model requires less computational complexity than multilayer perceptron. The ability of the random forest model to handle non-linear relationships and its resistance to overfitting can be one of the main reasons for its robust performance [43].

Statistical models like simple exponential smoothing and Croston’s method have performed exceptionally well and have outperformed strong machine learning models like XGBOOST. XGBOOST has given higher RMSE values, which resulted in the lowest rank among all the models. A primary reason for this underperformance is XGBOOST’s failure to capture seasonal patterns effectively. While models like XGBOOST can handle complex (non-linear) data interactions, they struggle with time-sequence datasets due to inefficiencies in capturing temporal dependencies [44]. Unlike MLP and Random Forest, which can model non-linear trends and periodic fluctuations, XGBOOST lacks inherent mechanisms to recognize long-term seasonal variations unless explicitly engineered. This limitation led to significant forecasting errors, particularly for products exhibiting cyclic demand patterns.

Smaller organizations that lack the necessary resources for developing machine learning models can use simple exponential smoothing and Croston’s method, despite their failure to yield better results than other machine learning models. The study’s results suggest that if one must choose between the two statistical models, one should select Croston’s method.

In conclusion, the findings suggest that organizations could significantly benefit from investing in machine learning approaches. However, it’s crucial to consider the trade-offs between accuracy and processing resources. While MLP has shown superior performance, it requires substantial computational resources.

## Conclusions

Accurate demand forecasting is essential for businesses to achieve rapid growth and minimize losses. The study aimed to analyze and contrast statistical and machine learning models for demand forecasting in the retail sector of Bangladesh. The study demonstrates that machine learning models, specifically multilayer perceptron and random forest, have exhibited outstanding performance and emerged as the top performers. However, traditional statistical models are not significantly lagging behind. Simple exponential smoothing and Croston’s method have performed better than XGBoost model. The analysis clearly demonstrates that the accuracy of the models is individual to each product and relies on the characteristics of the data. It would be prudent to select the forecasting models based on the sales data. In the end, the individual requirements and constraints of the retail environment will determine which of these tactics is the most effective. The study’s methodology and findings are applicable across multiple sectors for supply chain optimization, inventory management, and market trend analysis. Future research should look into ensemble techniques that integrate statistical and machine learning models. Furthermore, integrating larger datasets and real-time data streams could significantly improve model performance.

## Limitations

The study is inherently limited in some aspects that may have impacted the results and the scope of the findings. The quality and size of the dataset play a crucial role for the accuracy of the forecasting models. The dataset used in this study is small, which created significant challenges for training the machine learning models. Furthermore, a small dataset cannot demonstrate the entirety of consumer behavior, seasonal trends, and the effects of promotional offers, which impacts the generalizability of the study. The authors of the study had to face significant challenges while collecting sales data of a Bangladeshi restaurant. To overcome this issue, restaurants in Bangladesh need to adopt a digital sales recording system and keep track of all their promotional offers.

The researchers have chosen some model for comparative analysis in this study, but comparing all available models isn’t a possible task. Some models may yield better results than those used in the study. Moreover, the researchers couldn’t explore all the parameter configurations for the forecasting models due to time and resource constraints. Specific assumptions made during the methodology and data processing stage could also compromise the quality of the work. By recognizing these limitations, future researchers can accurately comprehend the study’s findings and identify opportunities to improve demand forecasting in Bangladesh’s retail sector.

## Supporting information

S1 Table Underlying data for calculating Average MAPE of different forecasting methods. (PDF)
