# Supplementary material for: A comparative study of various statistical and machine learning models for predicting restaurant demand in Bangladesh
Source: PLoS One. 2025 Jun 4;20(6):e0325449. doi: 10.1371/journal.pone.0325449 (PMC12136425; doi:10.1371/journal.pone.0325449)
Supplement: S1 Table — (PDF) [file pone.0325449.s001.pdf]

S1 Table.

	Statistical Model		Machine Learning Model		
Product	Simple Exponential Smoothing	Croston Method	XGBoost	Random Forest	Multilayer Perceptron
Noodles	39.71	38.52	45.05	38.01	35.55
Chicken	48.26	49.20	94.10	81.08	77.81
Hot Coffee	53.60	45.85	45.81	45.55	33.69
Rice	35.62	31.15	37.36	31.33	26.60
Soup	54.87	48.13	58.62	66.19	56.33
Average MAPE	46.41	42.57	56.17	52.42	46.00
